# Comparing the Effects of Low-Protein and High-Carbohydrate Diets and Caloric Restriction on Brain Aging in Mice

**DOI:** 10.1093/geroni/igaa057.3103

**Published:** 2020-12-16

**Authors:** Devin Wahl, David Raubenheimer, Rafael de Cabo, David Sinclair, Stephen Simpson, Samantha Solon-Biet, David Le Couteur

**Affiliations:** 1 Colorado State University, Fort Collins, Colorado, United States; 2 University of Sydney, Sydney, New South Wales, Australia; 3 National Institute on Aging, Bethesda, Maryland, United States; 4 Paul F. Glenn Center for Biology of Aging Research at Harvard Medical School, Boston, Massachusetts, United States; 5 University of Sydney, Camperdown, New South Wales, Australia

## Abstract

The Geometric Framework for Nutrition (GFN) has revealed that ad-libitum low-protein, high-carbohydrate (LPHC) diets improve cardiometabolic health and extend lifespan in rodents, but it is not known whether these diets are also beneficial for brain health. Here, we utilized previous results from GFN studies and compared hippocampus biology and memory in mice subjected to 20% calorie restriction (CR) or provided ad-libitum access to several LPHC diets. RNA expression in the hippocampus of 15-month-old mice were similar between mice fed CR and LPHC diets. Nutrient-sensing proteins, including SIRT1, MTOR, and PGC1-alpha, were also influenced by diet; however, the effects varied by sex. CR and LPHC diets were associated with increased dendritic spines in dentate gyrus neurons. Mice fed CR and LPHC diets had modest improvements in the Barnes maze spatial recognition memory paradigm and novel object recognition test. LPHC diets recapitulate some of the benefits of CR on brain aging. Part of a symposium sponsored by the Nutrition Interest Group.

